# Joint Congestion Control and Resource Allocation in Cache-Enabled Sensor Networks

**DOI:** 10.3390/s19132961

**Published:** 2019-07-05

**Authors:** Yuan Ren, Guangyue Lu, Changyin Sun

**Affiliations:** Shaanxi Key Laboratory of Information Communication Network and Security, Xi’an University of Posts and Telecommunications, Xi’an 710121, China

**Keywords:** beamforming, congestion control, resource allocation, cache-enabled sensor networks, successive convex approximation, Internet of Things

## Abstract

In this paper, we investigate the optimal beamforming design to achieve joint congestion control and energy-efficient resource allocation in cache-enabled sensor networks. The network of interest works in the time-slotted mode. The dynamic buffering queue for each node is introduced to reflect the degree of network congestion and service delay. Then, a time-averaged sum rate maximization problem is proposed under the constraints of queue stability, instantaneous power consumption, average power consumption, and the minimum quality of service requirements. By introducing the method of Lyapunov optimization, the importance of buffering queue backlogs and sum rate maximization can be traded off, then the original queue-aware and time-averaged optimization problem is transformed into a weighted sum rate maximization problem at each time slot. It can be further converted into a second-order cone-programming problem by successive convex approximation, which is convex and can be efficiently solved by off-the-shelf solvers. Numerical results validate that wireless caching can greatly relieve the network congestion by reducing the buffering backlogs, and show that the proposed scheme can trade off the average queue length and time-averaged sum rate by selecting different control parameters.

## 1. Introduction

In recent years, the data traffic generated by mobile users has experienced explosive growth [1]. Along with the development of mobile networks, the Internet of Things (IoT) and other wireless techniques are expected to bring us a wide variety of mobile applications and even more mobile traffic will be generated [2,3]. Notably, it is predicted that most of the traffic will result from the multimedia video services, which require higher network throughput and stricter network latency. To meet these unprecedented traffic demands and challenges, the standardization process of the fifth-generation (5G) network is accelerated in aspects of the network capacity and latency [4,5]. However, the limited capacity of backhaul links becomes the bottleneck of the wireless networks for large-scale video transmissions. Moreover, much data traffic is produced minute by minute, and there are many repeated contents among it, which will be transmitted more than once during the traffic-peak time periods. Confronting such a severe situation, proactive caching is regarded as one of the most promising techniques in 5G communication system to effectively alleviate the severe backhaul burden and improve the service delay, which has drawn tremendous attention from the industries and academia [6,7,8,9].

Proactive caching refers to the technique which prefetches popular video files to the local cache of base station/cluster head (CH) via backhaul links during traffic-free periods [10]. Currently, there are many caching schemes, which can be roughly divided into two categories, namely coded caching [11,12] and uncoded caching [13,14], and both of them can significantly relieve network burden and reduce service delay. Caching has shown great potentials in heterogeneous networks [15], device-to-device (D2D) communications [16], small cell networks [17,18] and cloud radio access networks (C-RANs) [19,20] to make the wireless networks perform in an energy-efficient manner.

With caching in sensor networks, the repeated end-to-end content deliveries can be avoided. The sensor nodes with limited battery will consume less power for data transmission, especially for data re-transmission for loss recovery [21]. Thus, the life span of the entire sensor network can be prolonged. The caching schemes have been investigated in the literature. Specifically, Sun et al. [22] proposed a novel energy-aware and latency-guaranteed dynamic resource caching strategy. The cache equipment was able to cache suitable data resources and the energy savings from servers were maximized in the meanwhile. In [23], they also proposed the re-cache and re-allocation scheme for popular data resources among load-unbalanced cache equipment to improve the delay performance. Though proactive caching can relieve the traffic burden to a certain degree, it is of great necessity and importance to observe the fluctuation of data traffic to relieve network congestion in the time periods of traffic surge. However, the current research ignored the time-varying characteristic of the dynamic traffic changes in the cache-enabled networks. Moreover, the issues of network congestion and service delay were not considered in a long-term observation, which were usually studied in an analytical manner based on the theory of stochastic geometry, e.g., [24,25].

In this paper, we study the optimal beamforming design to achieve joint congestion control and resource allocation in cache-enabled sensor networks. We propose a centralized scheme to solve this problem. Compared to this centralized method, the distributed approach can also be adopted to solve the joint optimization problems, such as those proposed in [26,27] to solve the joint problem of uplink power control and transmission rate allocation. However, this distributed approach is not appropriate in the considered downlink beamforming design. This is because the joint information of nodes is needed [13,14,28], and the battery and computational capacity of the sensor nodes, rather than the CH, is limited [29]. The degree of network congestion and service delay can be reflected by the length of dynamic buffering queue for each node, and it is obvious that smaller queue length will lead to smaller service delay and less network congestion. Then, under the constraints of queue stability, instantaneous/average power consumption, and minimum quality of service (QoS) requirements, the queue-aware and time-averaged sum rate optimization problem is proposed to improve the QoS provided to end nodes in the long term. The main contributions of this paper are summarized in the following aspects.

- By selecting different control parameters, control decisions are made to assign different importance levels to network congestion and sum rate maximization. In the case with severe traffic burden, more importance is allocated to network congestion control; while there is little congestion, the network operators will pay more attention to improve the sum rate performance.
- We observe the time-varying characteristic of the traffic fluctuations in a long time period instead of the instantaneous observation. The traffic queues of mobile nodes are established to reflect the congestion conditions of the entire network, which will be beneficial for network operators to monitor the dynamic network conditions and make the proper control decisions.
- The method of Lyapunov optimization is introduced to transform the proposed time-averaged maximization problem into a weighted sum rate maximization problem at each time slot. Then this problem is further converted into a second-order cone-programming (SOCP) problem via successive convex approximation (SCA), which owns lower computational complexity and can be efficiently solved.

The rest of this paper is organized as follows. In Section 2, we present the system model and formulate the time-averaged sum rate maximization problem. Then, the optimization algorithm based on Lyapunov optimization and SCA is proposed in Section 3. Simulation results are presented in Section 4, and this paper concludes in Section 5.

## 2. System Model and Problem Formulation

In this section, we present the system model, including the physical channel model, video cache model, and dynamic queue model. Then a queue-aware and time-averaged sum rate maximization problem is formulated.

### 2.1. Physical Channel Model

**Figure 1:**
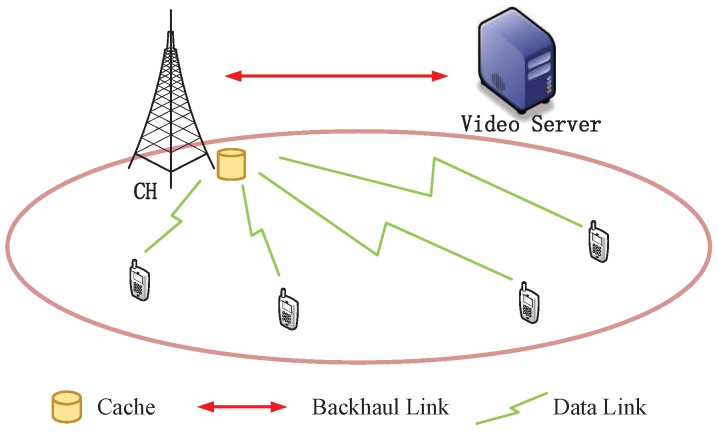
The CH with local cache capacity can provide *K* nodes with on-demand video services. If the required videos are cached in the local storage, the CH will directly serve the nodes with its local cache. Otherwise, the CH will deliver the required videos from the video server via backhaul links.

We consider a cluster with a CH to provide the on-demand video services to *K* single-antenna nodes, as shown in Figure 1. The cache-enabled sensor network works in the time-slotted mode, where the time slot is indexed by $t\in \left\{1,2,3,\ldots \right\}$. The CH is equipped with $R_{T}$ transmit antennas, and its transmit power is $P_{T}$. In each time slot, a block Rayleigh fading channel is considered. Let $h_{k}\left(t\right)\in C^{R_{T}\times 1}$ denote the channel from the CH to the *k*-th node. Define $w_{k}\left(t\right)\in C^{R_{T}\times 1}$ the beamforming vector from the CH to the *k*-th node at time slot *t*. The received signal for the *k*-th node can be written as

(1) $$\begin{array}{cc} y_{k}\left(t\right) & =h_{k}^{H}\left(t\right)w_{k}\left(t\right)x_{k}\left(t\right)+\sum_{n=1,\;n\neq k}^{K}h_{k}^{H}\left(t\right)w_{n}\left(t\right)x_{n}\left(t\right)+n_{k}\left(t\right), \end{array}$$

where $x_{k}\left(t\right)$ is the required data symbol of the required video file with unit power, and $n_{k}\left(t\right)$ is the additive white Gaussian noise following $\mathrm{CN}\left(0,\;\sigma_{k}^{2}\right)$. Based on the received signal, the signal-to-interference-plus-noise ratio (SINR) of the *k*-th node can be shown as

(2) $${\mathrm{SINR}}_{k}\left(t\right)=\frac{{\left|h_{k}^{H}\left(t\right)w_{k}\left(t\right)\right|}^{2}}{\sum_{n=1,\;n\neq k}^{K}{\left|h_{k}^{H}\left(t\right)w_{n}\left(t\right)\right|}^{2}+\sigma_{k}^{2}}.$$

Therefore, the rate of the *k*-th node can be expressed as

(3) $$R_{k}\left(t\right)=\log_{2}\left(1+{\mathrm{SINR}}_{k}\left(t\right)\right).$$

### 2.2. Video Cache Model

Note that the CH is equipped with local storage capacity, and can cache the most popular video files in advance. Assume that there are *M* files in the video server. Owning to the limited cache size, the CH can only caches a fraction, i.e., η, of these video files, which means the ⌊ηM⌋ most popular videos are locally cached. At the beginning of each time slot, each node is supposed to request only one video file, and the CH will collect these demands periodically. All videos are sorted in the descending order of popularity where more popular videos are ranked with smaller indices. The probability of video requests of mobile nodes follows the Zipf’s law [30]. To this end, the probability of the video file requested by the *k*-th node, i.e., $m_{k}$, can be obtained as

(4) $$p_{m_{k}}=\frac{i_{m_{k}}^{-\alpha }}{\sum_{j=1}^{M}j^{-\alpha }},\;\;i_{m_{k}}\in \left\{1,\;2,\ldots ,\;M\right\},$$

where $i_{m_{k}}$ is the index of video popularity order requested by the *k*-th node and α is the skewness parameter. In general, a larger α indicates most node requests can be satisfied by a few video files. Moreover, the time scale of video update is much larger than that of video delivery, e.g., 2–3 h for news with short videos and one week for movies [31], so this issue is not considered in this paper.

### 2.3. Dynamic Queue Model

In the considered cache-enabled sensor network, we intend to supervise and control the data traffic generated from the on-demand video services, such that the network congestion can be avoided, and the service delay is reduced. It is a common sense that longer buffering queue length will lead to larger service delay and more serious network congestion. Therefore, it is of vital significance to control the length of buffering data traffics of each node to achieve a stable and robust cache-enabled sensor network. If the network is in heavy traffic load, sufficient attention should be paid to the network congestion and proper decisions should be made to reduce the traffic backlogs. In this context, traffic buffering queues are introduced to reflect the fluctuation of data traffic, where the traffic arrival rate is denoted as $A_{k}\left(t\right)$ and the departure rate is $R_{k}\left(t\right)$ as defined in (3). Please note that the CH is equipped with local storage capacity, thus the traffic arrival rate $A_{k}\left(t\right)$ will be zero if the requested video file $m_{k}$ can be obtained locally. Then the binary constant $c_{m_{k}}$ is introduced to indicate whether the requested video $m_{k}$ is cached in the CH or not. Therefore, the arrival rate $A_{k}\left(t\right)$ can be obtained as

(5) $$A_{k}\left(t\right)=\left\{\begin{array}{cc} \log_{2}\left(1+\gamma_{k}\right), & c_{m_{k}}=0,\\ 0, & c_{m_{k}}=1, \end{array}\right.$$

where $\gamma_{k}$ is the target QoS requirement to fetch the requested video file from the video server through backhaul links for the *k*-th node. Then, at the $\left(t+1\right)$-th time slot, the traffic buffering queue for the *k*-th node can be denoted as

(6) $$Q_{k}\left(t+1\right)=max\left\{Q_{k}\left(t\right)-R_{k}\left(t\right),\;0\right\}+A_{k}\left(t\right).$$

The queue length in the subsequent time slot only depends on the arrival rate, departure rate, and queue length in the previous time slot. To describe the stability of these queues, we can resort to the following definition [32].

**Definition** **1.** *The discrete time process Q(t) is mean rate stable if*

(7) $$\lim_{t\to \infty }\frac{1}{t}E\left\{\left|Q(t)\right|\right\}=0.$$

*Though the actual buffering queues are non-negative, we regulate Q(t) in the form of absolute value so that this definition can be extended to the virtual queues whose values are likely to be negative.*

### 2.4. Problem Formulation

We aim to observe the queue stability of the cache-enabled network in the long term, and consider the time-varying characteristics of the multimedia data traffic in the meanwhile. Therefore, a queue-aware and time-averaged sum rate maximization problem is proposed, which can be shown as

(8a) $$\begin{array}{cc} \max_{\left\{w_{k}\left(t\right)\right\}}\; & \lim_{T\to \infty }\sum_{t=1}^{T}\sum_{k=1}^{K}\frac{1}{T}E\left\{R_{k}\left(t\right)\right\} \end{array}$$

(8b) $$\begin{array}{cc} s.t.\;\; & Q_{k}\left(t\right)\;\mathrm{is}\;\mathrm{mean}\;\mathrm{rate}\;\mathrm{stable},\;\forall k,\;t, \end{array}$$

(8c) $$\begin{array}{c} \sum_{k=1}^{K}{∥w_{k}\left(t\right)∥}^{2}\leq P_{T},\;\forall t, \end{array}$$

(8d) $$\begin{array}{c} \lim_{T\to \infty }\sum_{t=1}^{T}\sum_{k=1}^{K}\frac{1}{T}{∥w_{k}\left(t\right)∥}^{2}\leq P_{\mathrm{avg}}, \end{array}$$

(8e) $$\begin{array}{c} {\mathrm{SINR}}_{k}\left(t\right)\geq \gamma_{k},\;\forall k,\;t, \end{array}$$

where $P_{\mathrm{avg}}$ is the average power consumption. The stability of the buffering queues can be satisfied in constraint (8b). Constraint (8c) restricts the budget of instantaneous power consumption, while (8d) is the constraint of average power consumption observed in an infinite time period. Meanwhile, the minimum QoS requirements are guaranteed in the last constraint.

## 3. Congestion Control and Resource Allocation Optimization

In this section, the method of Lyapunov optimization is introduced to transform the original optimization problem into a weighted sum rate maximization problem at each time slot, where the control decisions can be made to trade off the importance of network congestion and sum rate maximization. Then, this problem is converted into an SOCP problem, which requires less computation effort and can be easily solved.

### 3.1. Lyapunov Optimization

Firstly, we construct a virtual queue related to the constraint (8d), which is shown in the following proposition.

**Proposition** **1.** *S(t) is a virtual queue with $E\left\{\left|S(0)\right|\right\}<\infty$. At time slot $\left(t+1\right)$, the virtual queue can be denoted as*

(9) $$S\left(t+1\right)=max\left\{S\left(t\right)-P_{\mathrm{avg}},\;0\right\}+P\left(t\right),$$

*where $P\left(t\right)=\sum_{k=1}^{K}{∥w_{k}\left(t\right)∥}^{2}$. If the virtual queue S(t) is mean rate stable, the constraint (8d) holds.*

**Proof.**  The details for the proof are provided in Appendix A. □

From Proposition 1, the constraint (8d) regarding to average power consumption is transformed into the issue of virtual queue stability. To this end, there are two kinds of queues, i.e., buffering queue $Q_{k}\left(t\right)$ and virtual queue S(t). Denote $\Xi \left(t\right)=(Q_{1}\left(t\right),\;Q_{2}\left(t\right),\ldots ,\;Q_{K}\left(t\right),\;S\left(t\right))$ the mixed queue vector of actual traffic queues and virtual queue. According to [32], the scalar Lyapunov function can be defined as

(10) $$L\left(\Xi \left(t\right)\right)=\frac{1}{2}\left\{\sum_{k=1}^{K}Q_{k}^{2}\left(t\right)+S^{2}\left(t\right)\right\}.$$

This function can roughly reflect the degree of network congestion. Specifically, if the value of Lyapunov function L(Ξ(t)) is low, it means that all the queue lengths are small, and the system is stable and robust. However, the value of this function will increase if there is at least one queue whose traffic backlog is large. Next, the concept of Lyapunov drift is adopted to force the Lyapunov function to a lower value, so as to obtain smaller queue length and buffering backlogs [32], which can be expressed as

(11) $$▵\left(\Xi \left(t\right)\right)=E\left\{L(\Xi (t+1))-L(\Xi (t))\right\}.$$

To jointly control the network congestion and maximize the time-averaged sum rate, we can resort to the drift-plus-penalty expression, as given by

(12) $$▵\left(\Xi (t)\right)-VE\left\{R_{k}\left(t\right)|\Xi \left(t\right)\right\},$$

where *V* is a non-negative control parameter to trade off the traffic backlogs and sum rate maximization. A larger *V* will put more emphasis on sum rate maximization, while a smaller *V* emphasizes more on network stability. So, we can make control decisions to achieve different requirements under different control parameters. It is obvious that a smaller value of this expression can achieve a smaller queue length and a larger sum rate. Then, the following proposition is recommended to present the upper bound of the drift-plus-penalty expression.

**Proposition** **2.** *At time slot t, under the observed queue state, the drift-plus-penalty expression of joint congestion control and sum rate maximization satisfies*

(13) $$\begin{array}{cc} ▵\left(\Xi (t)\right)-VE\left\{R_{k}\left(t\right)|\Xi \left(t\right)\right\} & \leq A+\sum_{k=1}^{K}Q_{k}\left(t\right)E\left\{A_{k}\left(t\right)-R_{k}\left(t\right)|\Xi \left(t\right)\right\}\\ & \;+S\left(t\right)E\left\{P\left(t\right)-P_{avg}|\Xi \left(t\right)\right\}-VE\left\{R_{k}\left(t\right)|\Xi \left(t\right)\right\}, \end{array}$$

*where A is a finite constant satisfying*

(14) $$\begin{array}{cc} A & \geq \frac{1}{2}E\left\{\sum_{k=1}^{K}R_{k}^{2}\left(t\right)+A_{k}^{2}\left(t\right)|\Xi \left(t\right)\right\}+\frac{1}{2}E\left\{P_{avg}^{2}+P^{2}\left(t\right)|\Xi \left(t\right)\right\}. \end{array}$$

**Proof.**  The details for the proof of this proposition are presented in Appendix B. □

To minimize the drift-plus-penalty expression, we can focus on minimizing the right-hand side of (13), which is the upper bound of the expression (12). To this end, the original optimization problem (8) can be transformed into

(15a) $$\begin{array}{cc} \min_{\left\{w_{k}\left(t\right)\right\}}\; & S\left(t\right)P\left(t\right)-\sum_{k=1}^{K}\left(Q_{k}\left(t\right)+V\right)R_{k}\left(t\right) \end{array}$$

(15b) $$\begin{array}{cc} s.t.\;\; & (8c),\;(8e). \end{array}$$

### 3.2. Weighted Sum Rate Maximization

After the transformation based on the method of Lyapunov optimization, the joint congestion control and time-averaged sum rate maximization problem has been converted into a weighted sum rate maximization problem. Then, this problem can be further transformed into an SOCP problem via SCA. For simplicity, we denote

(16) $$\begin{array}{c} a(t)=S(t),\;\forall t, \end{array}$$

(17) $$\begin{array}{c} b_{k}\left(t\right)=Q_{k}\left(t\right)+V,\;\forall k,t. \end{array}$$

Firstly, the slack variable $z_{k}\left(t\right)$ is introduced, satisfying $z_{k}\left(t\right)\leq \mathrm{SIN}R_{k}\left(t\right)$. Then, the approximation method used in [33,34] is adopted to obtain the lower bound of $\log_{2}\left(1+z_{k}\left(t\right)\right)$, which can be presented as

(18) $$\log_{2}\left(1+z_{k}\left(t\right)\right)\geq \theta_{k}\left(t\right)\log_{2}z_{k}\left(t\right)+\mu_{k}\left(t\right),$$

where

(19) $$\begin{array}{c} \theta_{k}\left(t\right)=\frac{\tilde{z_{k}}\left(t\right)}{1+\tilde{z_{k}}\left(t\right)}, \end{array}$$

(20) $$\begin{array}{c} \mu_{k}\left(t\right)=\log_{2}\left(1+\tilde{z_{k}}\left(t\right)\right)-\frac{\tilde{z_{k}}\left(t\right)}{1+\tilde{z_{k}}\left(t\right)}\log_{2}\tilde{z_{k}}\left(t\right). \end{array}$$

It is obvious that the equality holds in (18) when $\tilde{z_{k}}\left(t\right)=z{}_{k}\left(t\right)$. The selection of the point $\tilde{z_{k}}\left(t\right)$ will affect the optimal value of the optimization problem, so $\tilde{z_{k}}\left(t\right)$ can be updated by $z_{k}^{\left(n\right)}\left(t\right)$ iteratively where the superscript (n) means the optimal solution obtained in the *n*-th iteration. Then the slack variable $d_{k}\left(t\right)$ is introduced, which satisfies $\sqrt{\sum_{n=1,n\neq k}^{K}{\left|h_{k}^{H}\left(t\right)w_{n}\left(t\right)\right|}^{2}+\sigma_{k}^{2}}\leq d_{k}\left(t\right)$. After these manipulations, problem (15) can be converted as follows.

(21a) $$\begin{array}{cc} \min_{\left\{w_{k}\left(t\right),z_{k}\left(t\right),d_{k}\left(t\right)\right\}} & a\left(t\right)P\left(t\right)-\sum_{k=1}^{K}b_{k}\left(t\right)\left(\theta_{k}\left(t\right)\log_{2}z_{k}\left(t\right)+\mu_{k}\left(t\right)\right) \end{array}$$

(21b) $$\begin{array}{cc} s.t.\;\;\; & h_{k}^{H}\left(t\right)w_{k}\left(t\right)\geq \sqrt{z_{k}\left(t\right)}d_{k}\left(t\right),\;\forall k,t, \end{array}$$

(21c) ImhkHtwk(t)=0,∀k,t,

(21d) $$\begin{array}{c} \sqrt{\sum_{n=1,n\neq k}^{K}{\left|h_{k}^{H}\left(t\right)w_{n}\left(t\right)\right|}^{2}+\sigma_{k}^{2}}\leq d_{k}\left(t\right),\;\forall k,t, \end{array}$$

(21e) $$\begin{array}{c} (8c),\;(8e). \end{array}$$

As shown in (21c), the imaginary part of $h_{k}^{H}\left(t\right)w_{k}\left(t\right)$ is set to zero and this will have no effect on the optimal value of the optimization problem since phase rotations of beamforming vector $w_{k}\left(t\right)$ can bring out the same objective function while satisfying all constraints [35].

(1). Transformation of (21b). Please note that $\sqrt{z_{k}\left(t\right)}d_{k}\left(t\right)$ is not in the convex form, so it needs to be converted into the convex form. Referring to [35,36], the convex upper bound of $\sqrt{z_{k}\left(t\right)}d_{k}\left(t\right)$ can be shown as

(22) $$\sqrt{z_{k}\left(t\right)}d_{k}\left(t\right)\leq \frac{x_{k}}{2}d_{k}^{2}\left(t\right)+\frac{1}{2x_{k}}z_{k}\left(t\right),$$

when $x_{k}=\frac{\sqrt{z_{k}\left(t\right)}}{d_{k}\left(t\right)}$ the equality holds. Then (21b) can be approximated as

(23) $$\begin{array}{c} h_{k}^{H}\left(t\right)w_{k}\left(t\right)\geq \frac{x_{k}^{\left(n\right)}}{2}d_{k}^{2}\left(t\right)+\frac{1}{2x_{k}^{\left(n\right)}}z_{k}\left(t\right), \end{array}$$

and its second-order cone (SOC) form is

(24) $$\begin{array}{cc} \left||[\sqrt{2x_{k}^{\left(n\right)}}d_{k}\left(t\right);\;\left(h_{k}^{H}\left(t\right)w_{k}\left(t\right)-\frac{1}{2x_{k}^{\left(n\right)}}z_{k}\left(t\right)-1\right)]|\right| & \leq h_{k}^{H}\left(t\right)w_{k}\left(t\right)-\frac{1}{2x_{k}^{\left(n\right)}}z_{k}\left(t\right)+1, \end{array}$$

where

(25) $$x_{k}^{\left(n\right)}=\frac{\sqrt{z_{k}^{\left(n\right)}\left(t\right)}}{d_{k}^{\left(n\right)}\left(t\right)}.$$

(2). Transformation of (21d) and (8e). The SOC form of (21d) can be easily obtained as

(26) $$\begin{array}{c} \left|\right|\left[h_{k}^{H}\left(t\right)w_{1}\left(t\right);\ldots ;\;h_{k}^{H}\left(t\right)w_{k-1}\left(t\right);h_{k}^{H}\left(t\right)w_{k+1}\left(t\right);\ldots ;\;h_{k}^{H}\left(t\right)w_{K}\left(t\right)\right]\left|\right|\leq d_{k}\left(t\right). \end{array}$$

As for the constraint (8e), it can be approximated as

(27) $$h_{k}^{H}\left(t\right)w_{k}\left(t\right)\geq \sqrt{\gamma_{k}}d_{k}\left(t\right).$$

To this end, the optimization problem (15) has been transformed into an SOCP problem, as presented as follows

(28a) $$\begin{array}{cc} \min_{\left\{w_{k}\left(t\right),z_{k}\left(t\right),d_{k}\left(t\right)\right\}} & a\left(t\right)P\left(t\right)-\sum_{k=1}^{K}b_{k}\left(t\right)\left(\theta_{k}\left(t\right)\log_{2}z_{k}\left(t\right)+\mu_{k}\left(t\right)\right) \end{array}$$

(28b) $$\begin{array}{cc} s.t.\;\;\;\; & (8c),\;(21c),\;(24),\;(26),\;(27). \end{array}$$

The pseudo-code to solve the optimization problem (28) is presented in Algorithm 1. Note that the initial point of this algorithm is generated randomly until all constraints are satisfied. This has been validated a feasible method in practical simulations. 

**Algorithm 1:** The proposed iterative algorithm for solving (28)
1:Initialization: Set n=0, and find a point $\left(z_{k}^{\left(0\right)}\left(t\right),\;d_{k}^{\left(0\right)}\left(t\right),\;w_{k}^{\left(0\right)}\left(t\right)\right)$randomly that is feasible to problem (15).2:Repeat:3:Solve the SOCP problem (28) with $\left(z_{k}^{\left(n\right)}\left(t\right),\;d_{k}^{\left(n\right)}\left(t\right),\;w_{k}^{\left(n\right)}\left(t\right)\right)$and obtain the optimal point $\left(z_{k}^{*}\left(t\right),\;d_{k}^{*}\left(t\right),\;w_{k}^{*}\left(t\right)\right)$.4:Update $\left(z_{k}^{\left(n\right)}\left(t\right),\;d_{k}^{\left(n\right)}\left(t\right),\;w_{k}^{\left(n\right)}\left(t\right)\right)=\left(z_{k}^{*}\left(t\right),\;d_{k}^{*}\left(t\right),\;w_{k}^{*}\left(t\right)\right)$.5:Update $\theta_{k}\left(t\right)$ using (19), $\mu_{k}\left(t\right)$ using (20), $x_{k}^{\left(n\right)}$ using (25),then n=n+1.6:Until convergence or reach the requested number of iterations,   update a(t) using (16) and $b_{k}\left(t\right)$ using (17), then t=t+1.


### 3.3. Convergence and Complexity Analysis

At time slot *t*, supposing $\left(z_{k}^{\left(n\right)}\left(t\right),\;d_{k}^{\left(n\right)}\left(t\right),\;w_{k}^{\left(n\right)}\left(t\right)\right)$ is the optimal point in the *n*-th iteration, which is adopted as the starting feasible point in the (n+1)-th iteration. It is apparent that the optimal value of the objective function in the (n+1)-th iteration must be larger than or at least equal to that obtained in the *n*-th iteration for a maximization problem. Furthermore, the optimization problem (28) is bounded by the budget of transmit power consumption. Therefore, the convergence point is bound to be obtained after a sequence of non-decreasing points. After enough iterations, the difference of the optimal values between two adjacent iterations can be ignored. According to the conclusion presented in [36], the optimal solution also satisfies the Karush–Kuhn–Tucker (KKT) conditions of the optimization problem (21). After the transformation based on SCA, Algorithm 1 aims to solve the SOCP problem (28), which requires lower computation effort. To be more specific, by referring to [37], the maximum number of iterations is restricted by $O(\sqrt{5K+2})\ln\left(1/\zeta \right)$ when interior method is employed, where ζ is the accuracy threshold. The required computational cost for each iteration is bounded by $O(K^{3}\left(R_{T}^{3}+7R_{T}^{2}+16R_{T}+12\right)+\left(3K+1\right)\left(R_{T}+2\right))$.

## 4. Simulation Results

In this section, the numerical results are presented. In our simulations, we consider a small-scale sensor network, where one CH serves six nodes. This is because serving too many nodes for a CH will lead to unbearable computational complexity [20], and thus the QoS may not be guaranteed. When there are multiple nodes to be served, they can be divided into many clusters. Then, the beamforming design for each cluster can be realized by the same methods presented in this paper. MATLAB is used as a simulation tool, and SeDuMi is employed as an optimization solver [38]. Suppose the channels are independent and identically distributed with elements following the complex Gaussian distribution with zero mean and unit variance, i.e., $\mathrm{CN}\left(0,\;1\right)$ at each time slot. Assume that there are M=1000 video files in the video server and the skewness parameter α is set to 1 unless stated otherwise. The values of simulation parameters are summarized in Table 1.

**Figure 2:**
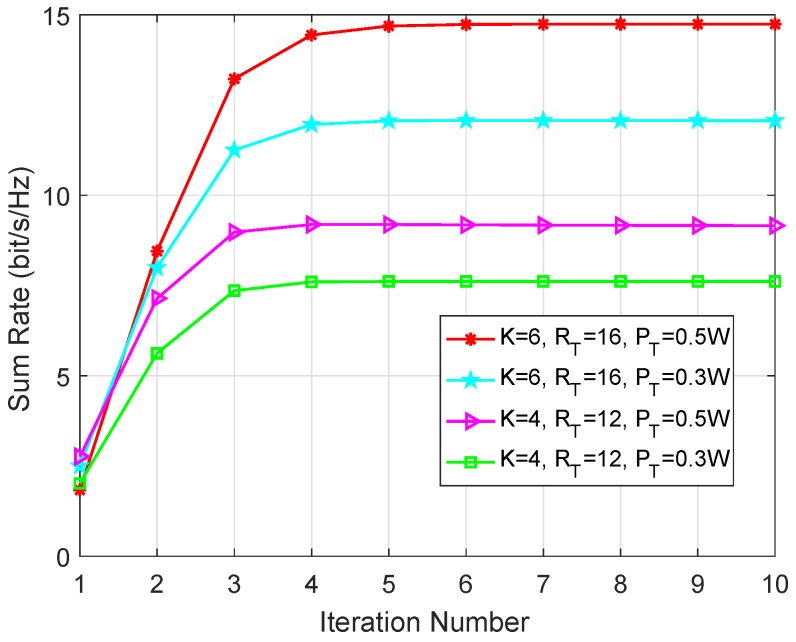
The convergence property of Algorithm 1.

Convergence Property of Algorithm 1: It is worth noting that the convergence property is validated in a random time slot. As shown in Figure 2, the proposed algorithm to solve the weighted sum rate maximization problem can converge to the local optimal point within a few number of iterations. So, Algorithm 1 shows great potentials in practical implementations and can be extended to the large-scale cache-enabled sensor networks to achieve joint congestion control and time-averaged sum rate maximization in an energy-efficient manner.

**Figure 3:**
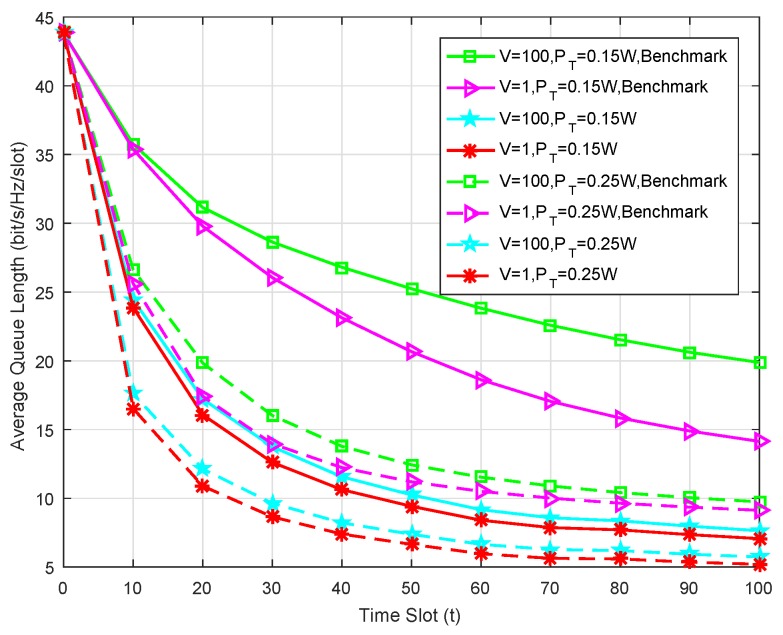
Average queue length at the observed time slot for K=6 and $R_{T}$ = 16.

Analysis of Average Queue Length: In Figure 3, we present how the average queue length changes in the observed time slots under the given control parameter *V*, where the average queue length is defined as $\bar{Q}\left(t\right)=\frac{1}{t}\sum_{\tau =1}^{t}\sum_{k=1}^{K}Q_{k}\left(\tau \right)$. Here the benchmark scheme for comparison is the CH without cache capacity. Firstly, it can be concluded that the proposed caching scheme can greatly reduce the average queue length under the given control parameter and arrival rate. Secondly, a larger control parameter *V* will lead to a longer average queue length, which can be intuitively understood that more emphasis is placed on the sum rate maximization. Finally, all curves tend to the stable levels when the observed time period is long enough, which strongly validates the stable characteristic of the traffic buffering queues.

**Figure 4:**
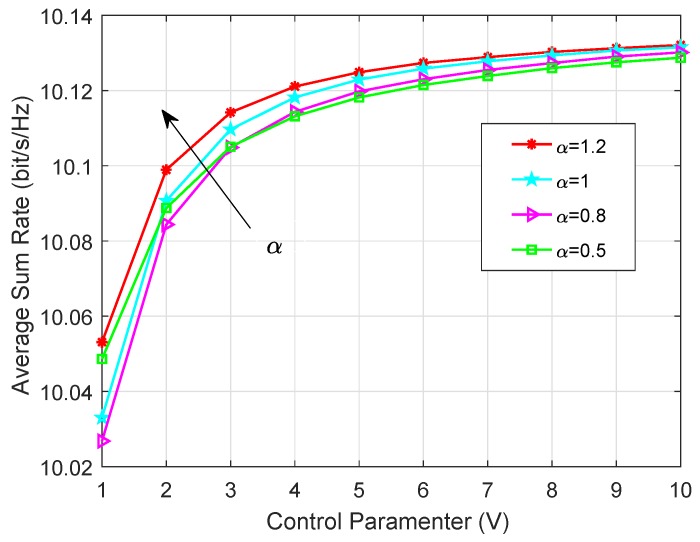
Time-averaged sum rate under different skewness parameter for K=6, $R_{T}=16$ and $P_{T}\;=\;0.2$ W.

Analysis of the Time-Averaged Sum Rate with Different Control Parameters: As shown in Figure 4, the average sum rate, defined as $\bar{R}=\sum_{t=1}^{T}\sum_{k=1}^{K}\frac{1}{T}E\left\{R_{k}\left(t\right)\right\}$, is increasing with the control parameter *V*, which can be explained that a larger *V* will emphasize more on the sum rate maximization and lead to the larger value of $\bar{R}$. Furthermore, under the given *V*, $\bar{R}$ will increase with the skewness parameter α. It is because that a larger α means that fewer videos will satisfy the majority of node requirements and more of them can be served by local cache, thus a smaller amount of arriving data traffics will be allowed to admit to the cache-enabled sensor networks, which can effectively relieve the network congestion and improve the sum rate of end nodes.

## 5. Conclusions

In this paper, we jointly considered the congestion control and resource allocation optimization in cache-enabled sensor networks. Firstly, we showed the system model and then proposed a time-averaged sum rate maximization problem. With the help of Lyapunov optimization, the original maximization problem was transformed into a weighted sum rate maximization problem at each time slot. Then, it was converted into an SOCP problem based on SCA. Simulation results demonstrated the fast convergence property of the proposed algorithm and the characteristic of dynamic queue stability under the proposed scheme. 

## Figures and Tables

**Table 1:** Values of Simulation Parameters.

Parameters	Values
*K*	6
$P_{T}$	0.2 W
$P_{avg}$	0.18 W
$R_{T}$	16
*M*	1000
η	0.1
α	1
$\alpha_{k}$	2 dB
$\sigma^{2}$	1

## Abbreviations

The following abbreviations are used in this manuscript:

IoT	Internet of Things
5G	Fifth Generation
CH	Cluster Head
D2D	Device-to-Device
C-RAN	Cloud Radio Access Network
QoS	Quality of Service
SOCP	Second-Order Cone Programming
SCA	Successive Convex Approximation
SINR	Signal-to-Interference-plus-Noise Ratio
SOC	Second-Order Cone
KKT	Karush–Kuhn–Tucker

## Appendix A

If the virtual queue S(t) is mean rate stable, it indicates that $\lim_{T\to \infty }\frac{1}{T}E\left\{\left|S(T)\right|\right\}=0$. Calculate the sum of (9) from t=0 to T−1, and we can get

$$\begin{array}{cc} \sum_{t=0}^{T-1}S\left(t+1\right) & =\sum_{t=0}^{T-1}\left[max\left\{S\left(t\right)-P_{\mathrm{avg}},0\right\}+P\left(t\right)\right]\\ & \geq \sum_{t=0}^{T-1}\left[S\left(t\right)-P_{avg}+P\left(t\right)\right], \end{array}$$

which is equivalent to

(A1) $$S\left(T\right)-S\left(0\right)\geq \sum_{t=0}^{T-1}P\left(t\right)-TP_{avg}.$$

Take expectation operation of (A1) and divide it by T→∞, then the left-hand side of (A1) can be shown as

$$\lim_{T\to \infty }\frac{1}{T}E\left\{\left|S(T)\right|\right\}-\lim_{T\to \infty }\frac{1}{T}E\left\{\left|S(0)\right|\right\}=0.$$

Therefore, $\lim_{{}_{T\to \infty }}\frac{1}{T}\sum_{t=1}^{T}P\left(t\right)-P_{\mathrm{avg}}\leq 0$ and the constraint (8d) is satisfied. This completes the proof of Proposition 1.

## Appendix B

Based on the definition of $Q_{k}\left(t+1\right)$, we can get

(A2) $$\begin{array}{cc} Q_{k}^{2}\left(t+1\right)= & {\left(max\left\{Q_{k}\left(t\right)-R_{k}\left(t\right),\;0\right\}+A_{k}\left(t\right)\right)}^{2}\\ \leq & Q_{k}^{2}\left(t\right)+R_{k}^{2}\left(t\right)+A_{k}^{2}\left(t\right)-2Q_{k}\left(t\right)\left(R_{k}\left(t\right)-A_{k}\left(t\right)\right). \end{array}$$

Taking sum of both sides in inequality (A2) for k=1,2,…,K, we can obtain

$$\begin{array}{cc} \sum_{k=1}^{K}\left(Q_{k}^{2}\left(t+1\right)-Q_{k}^{2}\left(t\right)\right) & \leq \sum_{k=1}^{K}\left[R_{k}^{2}\left(t\right)+A_{k}^{2}\left(t\right)-2Q_{k}\left(t\right)\left(R\left(t\right)-A_{k}\left(t\right)\right)\right]. \end{array}$$

Similarly, we can conclude that the virtual queue S(t) satisfies

$$\begin{array}{cc} S^{2}\left(t+1\right)-S^{2}\left(t\right) & \leq P_{\mathrm{avg}}^{2}+P^{2}\left(t\right)-2S\left(t\right)\left(P_{\mathrm{avg}}-P\left(t\right)\right). \end{array}$$

Therefore, the Lyapunov drift is bounded by

(A3) $$\begin{array}{cc} ▵(\Xi (t)) & =E\left\{L(\Xi (t+1))-L(\Xi (t))\right\}\\ & =\frac{1}{2}\left(\sum_{k=1}^{K}\left(Q_{k}^{2}\left(t+1\right)-Q_{k}^{2}\left(t\right)\right)+\left(S^{2}\left(t+1\right)-S^{2}\left(t\right)\right)\right)\\ & \leq \frac{1}{2}\sum_{k=1}^{K}\left(R_{k}^{2}\left(t\right)+A_{k}^{2}\left(t\right)-2Q_{k}\left(t\right)\left(R_{k}\left(t\right)-A_{k}\left(t\right)\right)\right)\\ & \;+\frac{1}{2}\left(P_{\mathrm{avg}}^{2}+P^{2}\left(t\right)-2S\left(t\right)\left(P_{\mathrm{avg}}-P\left(t\right)\right)\right). \end{array}$$

Subtract the term $VE\{R_{k}\left(t\right)|\Theta \left(t\right)\}$ in both sides of (A3), then it can be shown as the inequality (13) where *A* meets (14). Therefore, the proof of Proposition 2 is completed.
